# Acid-Free Electrochemical Regeneration of Sandrose-like Aluminum Layered Double Hydroxide Electrodes for Selective Lithium-Ion Recovery in Mixed Ion Solution

**DOI:** 10.1021/acssuschemeng.5c08261

**Published:** 2025-10-31

**Authors:** Cansu Kök, Pablo Vega Hernández, Jean G. A. Ruthes, Oliver Janka, Antje Quade, Volker Presser

**Affiliations:** † 28391 INM − Leibniz Institute for New Materials, Campus D2 2, 66123 Saarbrücken, Germany; ‡ Department of Materials Science & Engineering, Saarland University, Campus D2 2, 66123 Saarbrücken, Germany; § saarene − Saarland Center for Energy Materials and Sustainability, Campus C4 2, 66123 Saarbrücken, Germany; ∥ Inorganic Solid State Chemistry, Saarland University, Campus C4 1, 66123 Saarbrücken, Germany; ⊥ 28372 Leibniz Institute for Plasma Science and Technology, Felix-Hausdorff-Straße 2, 17489 Greifswald, Germany

**Keywords:** aluminum-layered double hydroxide, electrochemical regeneration, lithium ion recovery, selective adsorption, ion separation, lithium-ion extraction, intercalation materials

## Abstract

The demand for lithium production has seen a significant rise, with the growing electric vehicle and stationary battery markets requiring further development of sustainable and scalable extraction methods. Direct lithium extraction technologies have been developed to address potential shortages, with adsorption emerging as a key method due to its efficiency and low environmental impact. Given that Al­(OH)_3_ is already utilized as an adsorbent in various industrial applications, the practical importance of Al-based alternative systems for lithium ion extraction is increasing, yet lithium ion recovery requires harsh chemicals. In this study, we report a novel lithium extraction method combining chemical adsorption and electrochemical release using a synthesized aluminum layered double hydroxide (Al-LDH) material, developed under mild reaction conditions. The performance of the Al-LDH electrode was evaluated against a commercial Al­(OH)_3_ adsorbent. Comprehensive characterization using techniques such as X-ray diffraction, Fourier-transform infrared spectroscopy, and scanning electron microscopy revealed detailed insights into the crystalline structure, particle size distribution, and surface morphology of the materials. The Al-LDH electrode exhibited a lithium ion adsorption capacity, achieving an average chemical uptake of lithium ions of 57.6 mg/g. In contrast, lithium-ion uptake capacity for Al­(OH)_3_ was 1.0 mg/g over 15 cycles. Notably, this method operates under pH-neutral conditions, eliminating the need for harsh acidic or basic eluents. As a result, it prevents structural degradation and minimizes secondary pollution for potential future applications of lithium-ion recovery. The material’s layered structure selectively allowed lithium ion intake while blocking sodium ions, demonstrating its high selectivity and utility in lithium ion recovery processes. The integration of pH-neutral regeneration and high selectivity shows that Al-LDH electrodes as viable candidates for next-generation, green lithium extraction technologies.

## Graphical abstract

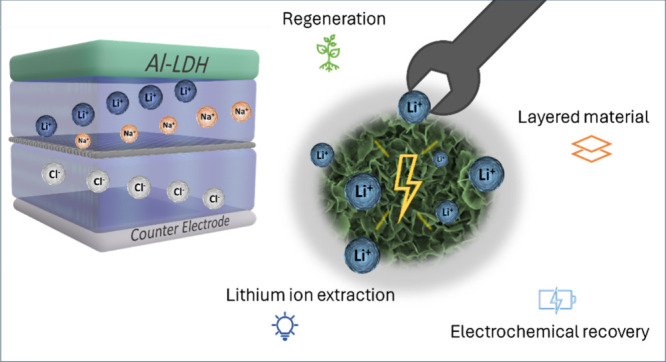

## 1 Introduction

The demand for lithium, strongly driven by battery production, is increasing from 32,000 tons in 2012 to a range of 850,000 to 1,200,000 tons by 2050.^1^ Recent reports show a 23% annual rise in lithium production, which climbed from 146,000 tons in 2022 to roughly 180,000 tons in 2023.^2^ The production of electric vehicles is a significant driving force for the growing demand for lithium-ion batteries.^3,4^ Around 14 million electric vehicles (EV) were purchased in 2023, and the global sales are projected to reach 17 million in 2024.^3^ Favorable policies, in addition to more positive consumer perception of electric mobility, are expected to further bolster EV sales to more than 40 million units by 2030.^3^ Accordingly, investment in electric vehicles and battery manufacturing has risen to around 500 billion USD.^3^ The corresponding increase in lithium consumption may deplete the currently available lithium resources,^4,5^ which come primarily in the form of lithium carbonate (Li_2_CO_3_), lithium oxide (Li_2_O), lithium hydroxide (LiOH), and spodumene (LiAlSi_2_O_6_) extracted from continental brines (Li_2_CO_3_) and hard-rock minerals (Li_2_O, LiOH, and spodumene).^4^

The rising demand for lithium and its forecasted scarcity from the most common sources (such as hard-rock minerals and brines) necessitate the development of efficient methods for removing lithium from the environment.^6^ Numerous commercial and laboratory-scale methods for direct lithium extraction (DLE) exist, including evaporation, direct precipitation, membrane processes, solvent extraction, sorption, and ion exchange.^7,8^ But these methods are hindered by high chemical consumption, cost, and scalability, and environmental impacts such as soil and water contamination, higher energy demands.^9^ It is important to prioritize the efficient and environmentally friendly recovery of lithium.^10,11^

Among the various DLE technologies, adsorption is distinguished as the most advanced method, currently at technology readiness level (TRL) 9/9, due to its high theoretical lithium uptake capacity and low energy consumption.^7,12^ Adsorption serves as a cost-effective technique for recovering lithium ions by selectively removing them from aqueous media. This process plays a crucial role in assisting a circular economy.^13^ In the sorption-based DLE process, lithium ions are separated from complex, multi-ion aqueous environments through the use of aluminum-based sorbents that exhibit a high degree of selectivity for lithium ions over other competing ions. These specialized sorbents bind lithium ions from the solution, effectively concentrating the lithium ions while leaving other elements behind. Once the lithium ions have been sorbed, they are subsequently released or desorbed by flushing the system with fresh water, which facilitates the recovery of lithium ions in a purified form. Sorption offers several advantages, including the absence of hazardous chemical reagents and acids, a low environmental impact, an extraction efficiency of over 90%, and suitability for commercial operation.^14,15^

While several organic and inorganic sorbents, such as lithium manganese oxides, titanium oxides, aluminum hydroxides, iron oxide hydroxides, lithium iron oxide, clay minerals, and zeolites have been examined, lithium aluminum hydroxides stand out as particularly promising due to the structure of gibbsite-type aluminum hydroxides (γ-Al­[OH]_3_).^1,9,16,17^ These materials offer significant advantages, including consistent adsorption efficiency, lower production costs, simple manufacturing, durable adsorption–desorption cycles, and easy regeneration.^18^ Lithium ions are attracted and adhere to the surface of gibbsite, which can occupy the central cavity of the hexagon formed by the six aluminum atoms in gibbsite; in contrast, larger alkaline earth metal ions are unable to do so due to steric hindrance.^7^ Positively charged layers are induced when lithium ions diffuse into these vacancies.^19^ Anions then intercalate into the interlayer to maintain charge balance. Dong et al. synthesized a porous LiAl-LDH material, intending to increase its adsorption capacity.^20^ Kotsupalo et al. showed that they obtained high stability after more than 200 cycles in a large-scale column system (25 t) with an aluminum-based layered adsorbent.^21^ Zhong et al. created two-dimensional Li/Al-LDHs with high lithium ion selectivity via coprecipitation to adsorb lithium ions at room temperature.^22^ The effect of salinity on lithium ion adsorption by LiAl-LDHs adsorbents was studied at various magnesium chloride concentrations to explore Li^+^/Mg^2+^ separation.^16^ Results showed that increasing magnesium chloride from 0 to 500 g/L raised lithium ion adsorption capacity from 0.6 to 3.0 mg/g.^16^

However, there are environmental concerns regarding the commonly applied methods for lithium ion release. The most common approaches are to dispose of the saturated sorbent or to chemically regenerate it, which generally requires a relatively high temperature (>50 °C) and produces a lower eluate LiCl concentration compared to ion exchange.^7,23^ Alternative regeneration methods, such as electrochemically assisted regeneration, have been proposed to circumvent the chemical treatment of the sorbents.^10,14,24,25^ Electrochemical ion separation enables energy and cost efficiency, a high extraction rate against selective ions, and minimal maintenance expenses.^11,25,26^ While the electrochemical regeneration of ions like Sb­(V) and Pb­(II) has been explored in the literature, to the best of our knowledge, no studies have investigated the electrochemical recovery of lithium ions using aluminum-based adsorbent.^14,27^

This study focuses on the room-temperature synthesis of Al-LDH material with the goal of advancing a synthesis method to form a material that can be used for selective electrochemical lithium ion recovery. By leveraging the architecture of Al-based adsorbent, we aimed to create an electrode with high lithium-ion selectivity, thereby improving its electrochemical reversibility and enhancing the critical desorption stage through a pH-neutral washing step. Al-based adsorbents can desorb lithium ions using pure water which creates hydration shells that enable lithium ion release without the use of acids, thus lowering operational expenditures by removing the need for neutralization and waste treatment expenses.^28^ Unlike traditional adsorption processes that typically require high temperatures and acidic conditions, this is a gentle yet effective method for achieving high lithium-ion selectivity and maintaining the electrode’s performance in continuous lithium-ion extraction processes. By testing the performance under various operating conditions, we aim to understand how process parameters influence lithium ion separation efficiency and electrode stability. A systematic study was conducted to evaluate the uptake-release capacity and purity performance of the Al-LDH electrode over 15 cycles, achieved through controlled voltage application. The findings reveal that the Al-LDH electrode not only exhibited superior adsorption and electrochemical release capacity but also demonstrated enhanced selectivity for lithium ions toward sodium ions, marking its potential as a highly effective material in this application. In addition, the implementation of a neutral pH washing step allowed the recovery of lithium ions that could not be recovered during the electrochemical process. This mild electrochemical regeneration approach eliminates the need for corrosive eluents, strengthens the method’s environmental compatibility, and positions Al-LDH as a promising material for sustainable lithium-ion extraction technologies.

## 2 Experimental Section

### 2.1 Synthesis of the Al-LDH Material

Al-LDH has been synthesized following previous work.^29^ 0.81 g LiOH·H_2_O (Sigma-Aldrich, >98.0%) and 2.4 g Al­(OH)_3_ (Sigma-Aldrich; reagent grade) were mixed in a two-neck flask with approximately 75 mass % of deionized (DI) water. The mixture was saturated with argon gas and stirred at room temperature for 48 h. Next, ion exchange was performed by gradually introducing HCl (37% Sigma-Aldrich) at an equivalent mole ratio to the base into the solution. The mixture was stirred for 1–2 h to ensure completion of the reaction while maintaining the solution’s pH above 5.5. After washing with deionized water and overnight vacuum filtration, the sample was subsequently ground into a granulated form.

### 2.2 Al-LDH Electrode Preparation

The Al-LDH electrode was prepared by mixing the as-prepared Al-LDH material, carbon black (Alfa Aesar, purity of 99.5 mass %), and polytetrafluoroethylene binder (60 mass % dispersion in water, Sigma-Aldrich) with a mass ratio of 8:1:1 in ethanol. The mixture is carefully ground to create a uniform paste. Then, the paste was rolled into a free-standing electrode with a thickness of 150–200 μm using a hot rolling cylinder press (MTI HR01, MTI Corp). The Al-LDH electrode was dried at 40 °C under vacuum overnight. Punched electrode discs with a diameter of 26 mm were used as working electrodes.

### 2.3 Material Characterization

The morphology was analyzed using scanning electron microscopy (SEM). A ZEISS GEMINI 500 microscope with an EDX detector from Oxford Instruments was used in the experiments. The samples were ground in a mortar, fixed on the copper foil, and transferred to the sample holder. The micrographs were collected at an acceleration voltage of 1 kV with a 20 μm aperture.

Transmission electron microscopy (TEM) was performed with a 2100F system (JEOL) at a voltage of 200 kV. A copper grid coated with lacey carbon was used as the sample holder. The samples were dispersed in ethanol via an ultrasonic bath, and then 10 μL was drop-casted onto the grids and dried for 24 h. Phase analysis of the material was conducted using X-ray diffraction (XRD) with a D8 Discover diffractometer (Bruker) equipped with a copper source (Cu Kα, 40 kV, 40 mA), a VANTEC two-dimensional detector covering a 20° 2θ angular range, a Göbel mirror, and a 1 mm point focus. The detector was repositioned 4 times, with each measurement lasting 1000 s, to encompass an angular range of 10–80° 2θ. All scans went through background subtraction and were normalized to (0–1). The samples were ground using a rotary motion in a mortar and transferred to the sample holder.

Additional X-ray diffraction measurements were carried out at room temperature on pulverized samples of all the compounds discussed, using a Bruker D8-A25 Advance diffractometer operating in Bragg–Brentano θ–θ geometry (goniometer radius: 280 mm). The instrument employed nonmonochromatic Cu *K*_α1,2_-radiation (λ = 154.0596 and 154.4425 pm). Diffraction data were collected over a 2θ range of 6–130°, with a step size of 0.013° 2θ and a total acquisition time of 1 h. A 12 μm Ni foil served as a *K*_β_ filter, and a variable divergence slit was installed on the primary beam side. On the secondary beam side, a LYNXEYE detector with 192 channels was utilized. The recorded data were evaluated using the Bruker TOPAS 5.0 software, employing both the fundamental parameter approach and the Rietveld method.^30^

Raman spectroscopy was conducted using a Renishaw inVia Raman microscope, which employed a neodymium-doped yttrium aluminum garnet laser operating at a wavelength of 532 nm. Each sample was placed on a glass slide, and spectra from 10 points were recorded with a 10 s exposure time and accumulated five times. The microscope employed a 50× magnifying lens with a numeric aperture of 0.5. Spectra were treated by cosmic ray removal and normalized to (0–1).

The nitrogen adsorption analyses at −196 °C were carried out by using a Quadrasorb IQ system (Anton Paar, formerly Quantachrome). Before each measurement, the samples were outgassed for 12 h at 100 °C under vacuum.^31^ The specific surface area was determined using the Brunauer–Emmett–Teller (BET) method, implemented through the Quadrasorb IQ software.^32^

Fourier-transform infrared spectroscopy (VERTEX 70v FT-IR Spectrometer, Bruker) was used to analyze the functional groups on the surface of the adsorbent material, including their bonding and interactions with adsorbates. The powders were directly analyzed under the lens without any additional modifications. The surface groups of the material were determined within the spectral range from 400 to 4000 cm^–1^.

The concentration of the feed and recovery solutions was determined with an inductively coupled plasma optical emission spectrometer (ICP-OES, ARCOS FHX22, SPECTRO Analytical Instruments).^33^ 2 mL of samples were collected before and after the experiment, and they were injected into the system with a 1 mL/min sample flow rate. We established the connection between each ion concentration and the corresponding ICP-OES signal intensity. The calibration was performed with mixed ion solutions containing known concentrations of 0.1, 0.5, 1, 2, 5, and 10 mM of LiCl, NaCl, KCl, CaCl_2_, and MgCl_2_.

X-ray photoelectron spectroscopy (XPS) was performed with the Kratos Axis Supra (Kratos Analytical). The photon source was a monochromatized Al K_α_ line. Survey spectra were acquired with an analysis area of 300 × 700 μm^2^ and 160 eV pass energy at 15 kV. The highly resolved measured spectra were acquired using a pass energy of 10 eV at 15 mA and 15 kV for Al 2p, C 1s, and O 1s scans. All spectra were analyzed using CasaXPS software (version 2.3.15). The peaks were calibrated to the aliphatic component in C 1s (285.0 eV). For measurements, the loose powders were spread onto the surface of a small piece (5 × 5 mm^2^) of carbon adhesive tape.^34^ The flat side of a freshly cleaned spatula was used to press the powders firmly into the tape. After removing the loose powder from the surface of the sample holder, the holder was inserted into the XPS load lock.

Thermogravimetric analyses (TGA) were carried out in air with a Netzsch TG-209–1 Libra from 0 to 1000 °C at a heating rate of 10 °C/min. Five milligrams were taken from each sample and put into the alumina crucibles (Ceramic, TG F1 Libra). Then, crucibles were fixed to the device. Experiments have been performed in an argon (99.9%) atmosphere.

### 2.4 Calculations

The sorption capacity was calculated according to eq 1:

$$\mathrm{sorption}\;\mathrm{capacity}=\frac{(c_{i}-c_{t})\times V}{m}$$ 1

where *c_i_* is the initial concentration, *c_t_* is the final concentration of the uptake-release process, *m* is the mass of the sorbent, and *V* is the volume of the solution.

$$\mathrm{capacity}\;\mathrm{regeneration}=\frac{\mathrm{Cation}\;\mathrm{release}\;\mathrm{capacity}}{\mathrm{Cation}\;\mathrm{uptake}\;\mathrm{capacity}}\times 100\%$$ 2

$$\mathrm{purity}=\frac{\Delta C_{\mathrm{Li}}}{\Delta C_{\mathrm{all}}}\times 100\%$$ 3

where Δ*C*_Li_ and Δ*C*_all_ are the concentration changes of lithium ions and all cations in the recovery solution.

The selectivity regarding the lithium-ion to sodium-ion ratio was calculated via:

$$\mathrm{selectivity}=\frac{\Delta C_{\mathrm{Li}}}{\Delta C_{\mathrm{Na}}}$$ 4

Here, Δ*C*_Li_ and Δ*C*_Na_ are the concentration changes of lithium and sodium ions in the feed solution with the unit of mmol/L, respectively.

Energy consumption was calculated according to eq 5:

$$\mathrm{energy}\;\mathrm{consumption}=\frac{E_{\mathrm{electrical}}}{\mathrm{cation}\;\mathrm{release}\;\mathrm{capacity}}$$ 5

where *E*_electrical_ is the consumed electrical energy during one cycle.

### 2.5 Electrochemical Characterization

For electrochemical measurements of the cell, discs were punched from the prepared Al-LDH sheets and used as the working electrode (26 mm). An oversized commercially available microporous activated carbon cloth (Kynol ACC-507-10) was used as the counter electrode (26 mm) in a flow-through electrochemical desalination cell.^35^ The electrodes were separated by a few layers of glass-fiber filters (GF/A, Whatman). The same cell setup was repeated for the Al­(OH)_3_ electrode. The electrodes’ capacity to store and release lithium ions was evaluated using galvanostatic charge/discharge with potential limitation (GCPL) of +1 to −1 V with a 2 h holding time, conducted with a BioLogic VMP-300 potentiostat/galvanostat in a climate chamber maintained at 25 ± 1 °C, and the pH of the solution was not adjusted. Anion exchange membranes (Fumasep, FAS-PET-130) were used to prevent the uptake of competing ions. The electrochemical cell experiment proceeded in three stages, using two different solutions.

Initially, a feed solution containing 10 mM NaCl and 50 mM LiCl was pumped through the cell for 24 h at a flow rate of 3 mL/min at zero applied potential, allowing the electrodes to chemically adsorb the ions. Afterward, the electrodes were rinsed for 30 min by pumping deionized water (purified using a Millipore Milli-Q lab water system) to remove any lithium ions and sodium ions that had not been adsorbed into the electrode material. Finally, a recovery solution of 10 mM KCl was pumped through the cell while applying the GCPL technique with a current of 0.1 A/g, with a 1 V limit to the potential difference between the electrodes. During this stage, the electrodes were expected to release the lithium ions into the recovery solution.

Two mL of aliquots of both solutions were collected before and after each step. These three stages constituted a cycle, and the process was repeated 15 times to assess the electrodes’ ion storage/release capacity. The amount of lithium ions adsorbed and released during each cycle was determined using inductively ICP-OES. The Al-LDH and Al­(OH)_3_ electrodes have been analyzed through chronoamperometry in 1 M LiCl solution. Measurements have been run for approximately 14 h at 0.8 V.

## 3 Results and Discussion

In this study, an aluminum-based layered oxide material has been developed for the adsorption and electrochemical regeneration of lithium. The material has been synthesized at room temperature, ensuring that the process was carried out under mild and controlled conditions.

X-ray diffraction was used to prove that a crystalline material has formed during synthesis and to clarify which compound has formed, as the Pearson database (release 2023/2024) lists three different structures (Figure 1A,B). Anhydrous LiAl_2_Cl­(OH)_6_, which crystallizes in the hexagonal crystal system with space group *P*6_3_/*mcm* and two hydrated forms, LiAl_2_Cl­(OH)_6_·H_2_O and LiAl_2_Cl­(OH)_6_·*n*H_2_O with *n* = 1.50 to 2.16.^36,37^ While the monohydrate crystallizes with space group *P*6_3_/*m* the higher hydrated forms adopt the monoclinic crystal system with space group *C*2/*m*.^38,39^ According to Rietveld analysis, the prepared compound is the monoclinic variant. The amount of crystal water could not be refined from the collected powder data. In addition, the preferred orientation along [001] is observed, which is expected in view of the layered structure. Some minor discrepancies remain between the refined model and the collected data, which can be attributed to disorder and partially occupied sites. A crystallite size of 8 ± 1 nm was deduced from Rietveld fitting. The scanning and transmission electron micrographs of the samples are shown in Figure 1C,D. In our comparative analysis, we noted that the Al-LDH material exhibits a distinctive sandrose-like flaky structure, which presents an interesting contrast to the Al­(OH)_3_ structure (Supporting Information, Figure S1A,B). This type of structure indicates that lithium ions will penetrate the material more effectively.^20^ The transmission electron micrographs present in Figure 1E–H further display the sandrose-like structure of the material, along with the overlaid hierarchical layers, which corroborate with the scanning electron micrographs and other reported Al-LDH materials.^40–42^ Furthermore, the transmission electron micrographs suggest the formation of aggregates of the Al-LDH particles with different sizes. Micrographs indicate structures with hollow spots, characteristic of nonrigid aggregates, which could positively impact sorption–desorption, allowing more electroactive sites to be reached when compared with Al­(OH)_3_ powder (Supporting Information, Figure S1C–E).

**Figure 1:**
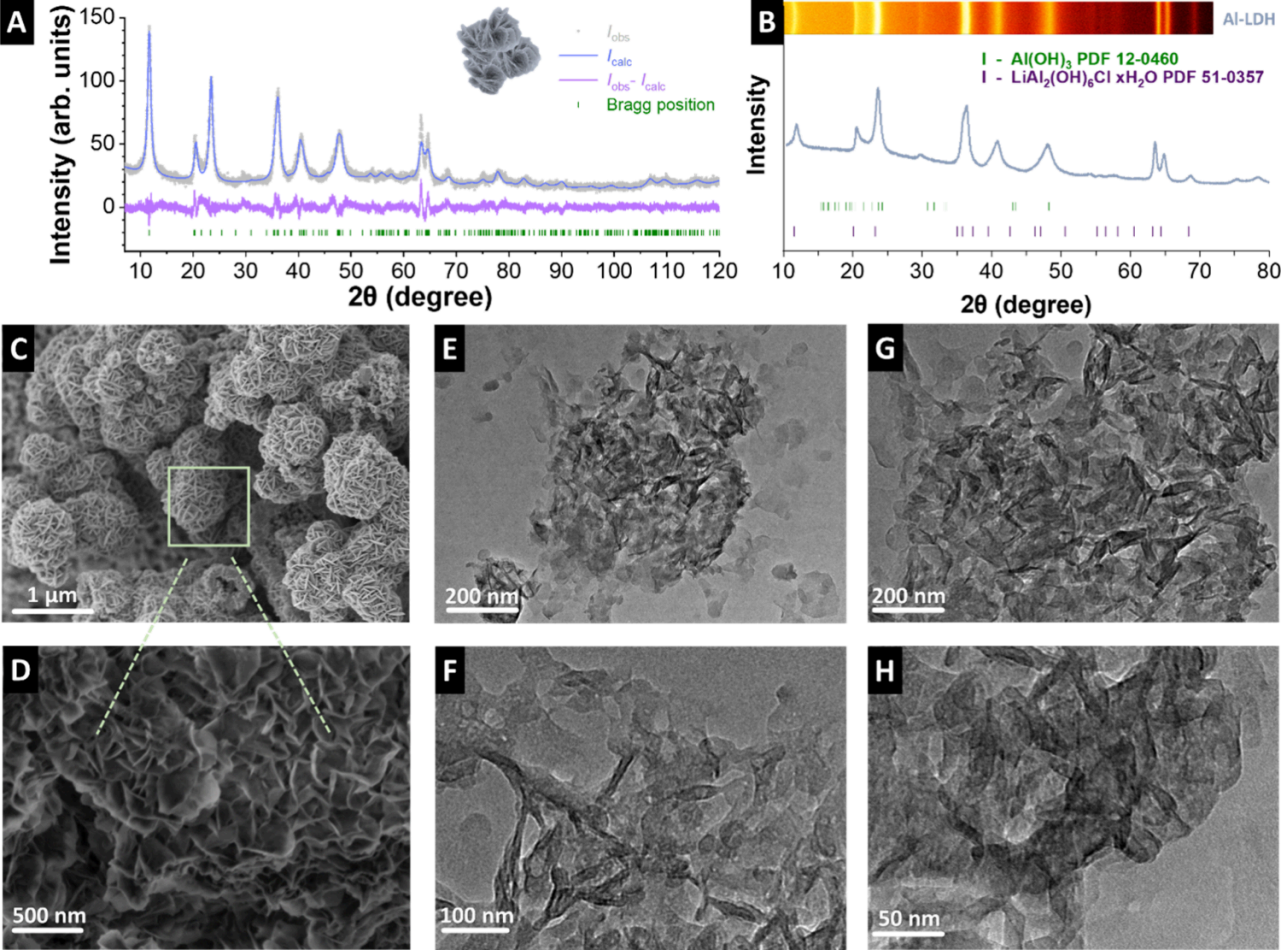
(A) X-ray diffractogram and associated Rietveld fitting of Al-LDH, (B) normalized X-ray diffractograms of Al-LDH, (C, D) scanning electron micrographs of Al-LDH in different magnifications, and (E–H) transmission electron micrographs of Al-LDH.

Surface area analysis was utilized to interpret parameters such as pore volume, specific surface area, and pore size. Analysis showed that the material has an average BET surface area of 60 m^2^/g (Figure 2A). In comparison, Al­(OH)_3_ shows a value of 23 m^2^/g for the BET specific surface area, which is lower than that of the Al-LDH material (Supporting Information, Figure S2). The noticeable increase in specific surface area and pore volume indicates more accessible sites for Faradaic reactions and double-layer formation.^43^ Based on the hysteresis loops of the materials by IUPAC, the Al-LDH material exhibits an H3 loop type.^44^ This type of hysteresis loop is typically associated with nonrigid aggregates of plate-like particles, as observed in the transmission electron micrographs. The layered structure of Al-LDH consists of positively charged metal hydroxide sheets with anions and water molecules located in the interlayer spaces. This configuration provides both high surface area and accessible channels for lithium ions to enter, interact, and be selectively adsorbed within the structure.^45^

**Figure 2:**
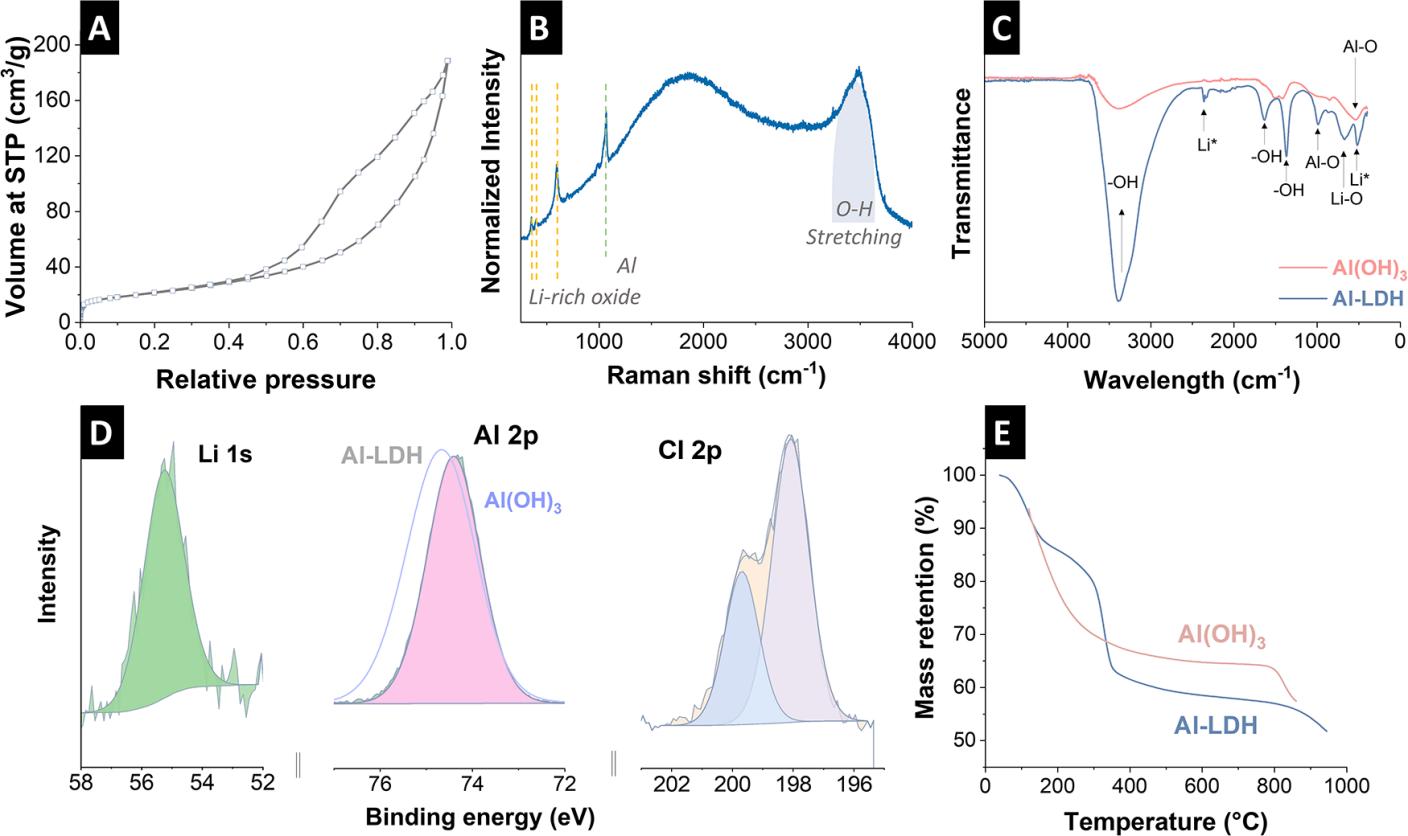
(A) Nitrogen gas sorption isotherm of Al-LDH at −196 °C (STP: standard temperature and pressure), (B) Raman spectrum of Al-LDH, (C) comparison of FTIR spectra of Al-LDH and Al­(OH)_3_, (D) X-ray photoelectron spectra of Al-LDH, (E) TGA results of the Al-LDH and Al­(OH)_3_ in Ar atmosphere.

Raman spectroscopy (Figure 2B) showed three apparent bands from lithium-rich oxides at 351, 396, 595 cm^–1^.^46^ The prominent vibrational mode observed at 595 cm^–1^ is primarily associated with the A_1g_ and E_g_ symmetries from rhombohedral group **R3** ***m***, respectively.^47^ Appeared peak in 1065 cm^–1^ is the characteristic band for the presence of Al.^48,49^ A typical bending mode ν_2_ near 1595 cm^–1^ from a single H_2_O molecule and 3320–3600 cm^–1^ range because of the O–H stretching band in the spectra.^50,51^

FTIR spectra were used to identify functional groups, with signals in the 2990–3600 cm^–1^ range attributed to the stretching vibrations of −OH groups and bands in the 1380–1635 cm^–1^ range corresponding to the bending vibrations of −OH groups. (Figure 2C).^13^ The bands at 538 and 990 cm^–1^ indicate the presence of the Al–O bond.^20^ The bands between 980 and 650 cm^–1^ can be attributed to the vibration of metal–oxygen bonding.^20^ Bands typically observed around 3300–3600 cm^–1^, corresponding to O–H stretching of Al­(OH)_3_.^52^

The chemical composition of the samples was also studied using XPS. According to the literature, the Al 2p signal of Al_2_O_3_ is positioned at a binding energy (BE) of 74.1 eV.^53^ However, a higher BE of 74.4 eV for Al 2p indicates the presence of a phase consisting of aluminum and lithium ions, which suggests the presence of the Al-LDH material (Figure 2D). Furthermore, the position of the Li 1s peak at a binding energy of 55.3 eV corresponds to the value published by Visser et al. for Li in Li-LDH.^54^ The Cl 2p peak is positioned at a binding energy of 198.1 eV. Zhong et al. found a similar binding energy for Cl 2p and interpreted it as indicating that more lithium chlorides were intercalated into Li/Al-LDHs (Figure 2D).^22^

Figure 2E compares the thermograms of Al-LDH and Al­(OH)_3_. Thermogravimetric analysis of the materials was performed in an argon atmosphere with temperatures ranging from 30 to 1000 °C. Al-LDH followed two steps of mass loss, including the initial weight loss due to the decomposition of physically adsorbed water molecules in the material.^55^ Our material decomposed at around 300 °C, and the total mass loss was 47% for the Al-LDH. The second mass loss can be attributed to the dehydroxylation of Al–OH.^56^

### 3.1 Electrochemical Cell Performance of the Al-LDH

The two-step process used by us to extract lithium ions is schematically illustrated in Figure 3A. Al-LDH enables effective lithium ion sorption due to its structure connected through hydrogen bonds, electrostatic interactions, and van der Waals forces.^7^ In the initial step, the adsorption process involves the chemical sorption of lithium ions. The second step involves the electrochemical desorption of lithium. This process was conducted by first washing the electrodes with deionized water and applying an electric charge to the electrochemical cell. Upon charging the cell, ions sorbed by the Al-LDH electrode are released back into the recovery solution, facilitating the regeneration of the electrode. The central cavities of the hexagonal Al­(OH)_3_ structure enable the penetration of lithium ions into the material.

**Figure 3:**
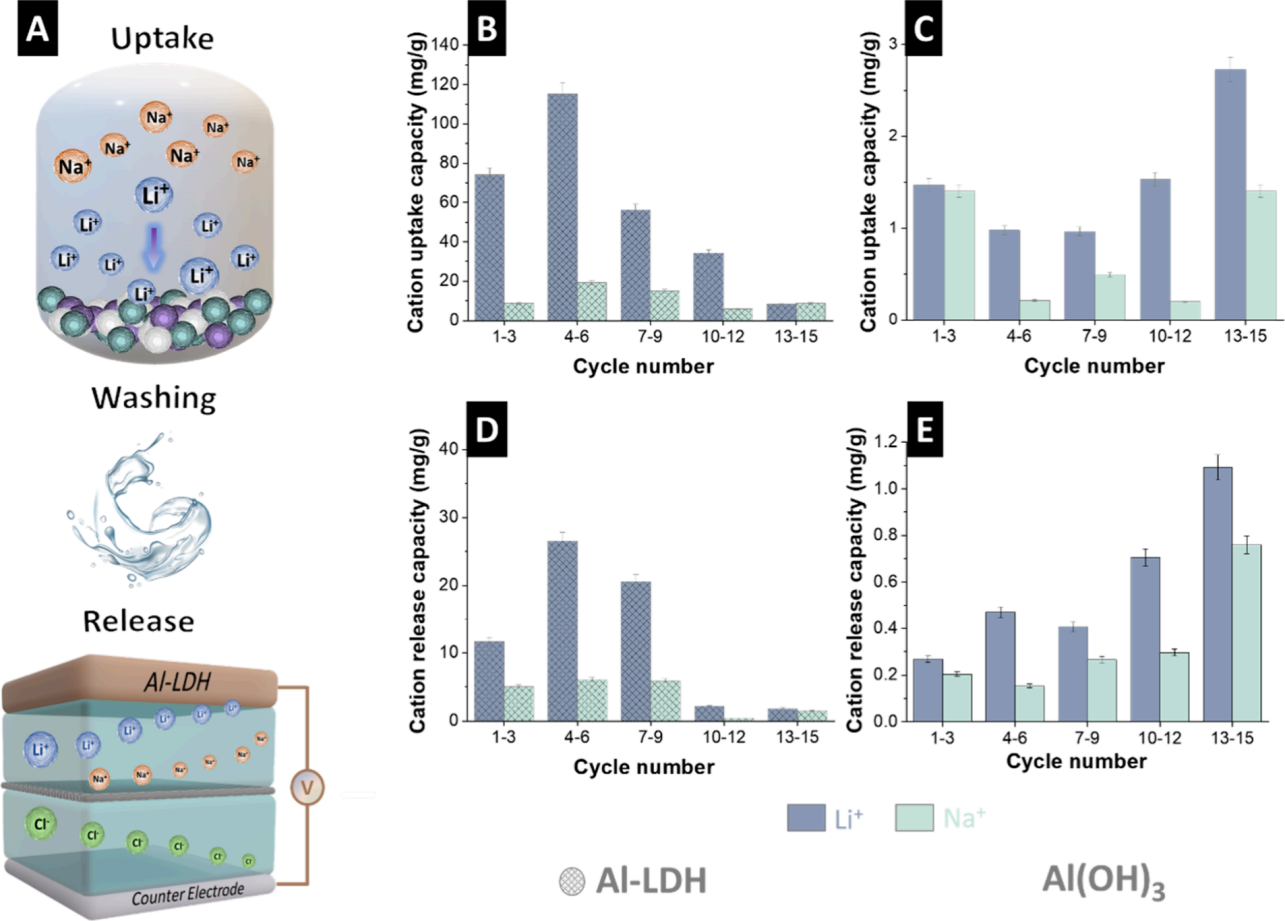
(A) Schematic representation of the chemical uptake, washing step, and electrochemical release process, (B) ion uptake capacity of the Al-LDH, (C) ion uptake capacity of Al­(OH)_3_, (D) ion release capacity of Al-LDH, and (E) ion release capacity of the Al­(OH)_3_.

The adsorption and desorption mechanisms of Al-LDH can be represented as follows:

$$\left[\mathrm{Vacancy}\right]\cdot {\mathrm{Al}}_{2}\mathrm{Cl}{\left(\mathrm{OH}\right)}_{6}+{{\mathrm{Li}}^{+}}_{\left(\mathrm{aq}\right)}\to \left[{\mathrm{LiAl}}_{2}\mathrm{Cl}{\left(\mathrm{OH}\right)}_{6}\right]$$ 6

$$\left[{\mathrm{LiAl}}_{2}\mathrm{Cl}{\left(\mathrm{OH}\right)}_{6}\right]+{H^{+}}_{\left(\mathrm{aq}\right)}\to \left[\mathrm{Vacancy}\right]\cdot {\mathrm{Al}}_{2}\mathrm{Cl}{\left(\mathrm{OH}\right)}_{6}+{{\mathrm{Li}}^{+}}_{\left(\mathrm{aq}\right)}$$ 7

Here lithium ions enter the vacancies in the material structure coordinated with chloride ions. The adsorption of lithium occurs with charge balancing using chloride ions, while desorption can be triggered by the external voltage that replaces lithium ions with protons.^57,58^

Electrochemical recovery of lithium ions was conducted in a solution containing 50 mM of lithium ions and 10 mM of sodium ions. Initially, for each cycle, the solution was pumped at a rate of 3 mL/min for 24 h to facilitate the adsorption of cations onto the electrode surface and then flushed with deionized (DI) water. ICP-OES showed that the uptake capacity of the lithium ions was much higher than that of sodium ions for the Al-LDH electrode (Figure 3B). After 15 cycles, the average uptake capacity for lithium was 57.6 mg/g, while the average uptake capacity for sodium was 12.5 mg/g. After pH-neutral washing, a voltage of 1 V was applied to the electrochemical cell for 2 h to facilitate the electrochemical release of cations. At the end of 15 cycles, the average lithium ion release capacity was calculated as 13 mg/g. The corresponding value for sodium ions is 3.8 mg/g (Figure 3D).

The average lithium ion uptake capacity for Al­(OH)_3_ was 1.0 mg/g, while for sodium ions, it was 0.4 mg/g (Figure 3C). We found that the average lithium-ion release capacity of the Al­(OH)_3_ was determined to be 0.6 mg/g. In contrast, the corresponding release capacity for sodium was calculated at 0.3 mg/g (Figure 3E). Comparative analysis revealed that the lithium ion release capacity of Al­(OH)_3_ was notably lower than that exhibited by the Al-LDH electrode.

To assess the relative contributions of sorption and charge to the release capacity following 15 cycles, the cell was flushed for 1 h with DI water after the chemical sorption stage in each cycle. The samples from the washing solution were collected to get back any ions that were lost during the uptake and release processes (Supporting Information, Figure S3).

Considering the material’s high adsorption capacity and its ability to undergo electrochemical regeneration, some ion loss was observed, which may reflect inherent trade-offs associated with the current system design. This loss is hypothesized to result either from the strong retention of ions within the material’s structure, preventing their complete electrochemical desorption, or from modifications occurring on the electrode surface. The binding affinity of different anions to the layered structure varies, following the order of Hofmeister lyotropic series: CO_3_^2–^ > HPO_4_^2–^ > HAsO_4_^2–^ > CrO_4_^2–^ > SO_4_^2–^ > OH^–^ > F^–^ > Cl^–^ > Br^–^ > NO_3_^–^. XPS analysis confirmed the presence of CO_3_^2–^ anions within the material’s layered framework (Supporting Information, Figure S4).^59,60^ Furthermore, postexperimental XRD characterization of the electrode suggests the formation of lithium aluminum carbonate hydroxide hydrate (Supporting Information, Figure S5). The high lithium ion selectivity of the layered hydroxide material likely arises from strong electrostatic or coordinative interactions between lithium ions and functional groups such as OH^–^. These strong interactions facilitate efficient ion capture but also increase the energy barrier for desorption, hindering ion release. These findings indicate the possibility of side reactions occurring during the process, as well as the inherent tendency of the adsorbent material to retain rather than fully release adsorbed ions. Moreover, post-mortem scanning electron micrographs of the electrode revealed significant structural degradation after 15 cycles (Supporting Information, Figure S6A-B).

The regeneration rate and purity were calculated to assess the reversibility of the electrodes and evaluate the efficiency of the lithium ion extraction process (Figure 4A,B). The regeneration ratio for Al-LDH generally ranged between 10 and 40%, which is consistent with the observed uptake-release capacity results. Despite Al-LDH demonstrating strong adsorption performance for lithium ions, it failed to fully release the captured ions during the electrochemical desorption stage. This asymmetry is attributed to the strong electrostatic interactions and coordination between lithium ions and the layered hydroxide host framework, which stabilizes the intercalated ions within the host framework.^19^ Additionally, potential structural rigidity and diffusion limitations within the interlayer spacing may impede ion mobility during the release phase.^58^ These findings suggest that while the material is highly effective for selective lithium ion capture, tuning interlayer spacing within the material structure and modifying host structure flexibility may be necessary to improve release efficiency.

**Figure 4:**
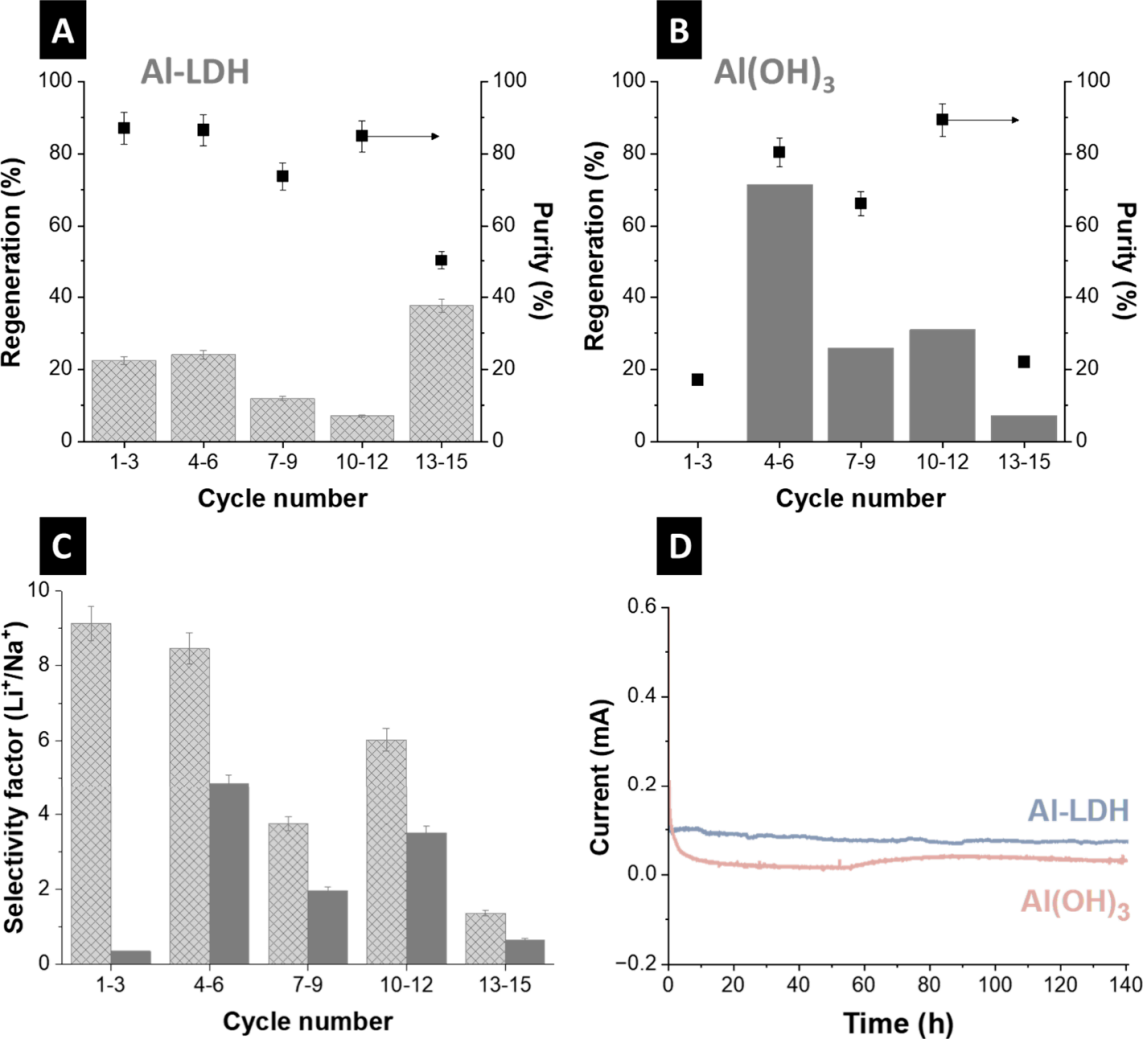
(A) Regeneration and purity of recovery solution for Al-LDH, (B) regeneration and purity of recovery solution for Al­(OH)_3_, (C) selectivity factor comparison of the feed solutions for Al-ALH and Al­(OH)_3_, (D) chronoamperometry measurements of Al-LDH and Al­(OH)_3_ electrodes vs Ag/AgCl in 1 M LiCl.

The purity of Al-LDH generally ranged between 70 and 90% and dropped to 50% in the last 3 cycles (Figure 4A). These values were inconsistent for the unmodified material Al­(OH)_3_ (Figure 4B). Figure 4C plots the lithium ions selectivity factor toward sodium ions, highlighting that the selectivity factor for the Al-LDH electrode is higher than that of the Al­(OH)_3_ electrode. The difference can be attributed to the selective ion transport properties of the Al-LDH electrode, in which the vacancies generated by the deintercalation of primordial lithium ions during the desorption process act as highly selective active sorption sites for lithium ions.^61^ Unlike Al-LDH, the AlOH_3_ electrode’s behavior suggests that it facilitates the passage of both lithium ions and sodium ions. The adsorptive nature of the Al­(OH)_3_ electrode implies that while it may be efficient for general ion uptake, it does not possess the discriminative capacity necessary for lithium-ion-specific applications. This limitation highlights the importance of electrode composition and structure in achieving targeted ion selectivity. The cell corresponding to Al-LDH had a higher current compared to Al­(OH)_3_, indicating the transport of cations. Chronoamperometry results provide that the current for Al–LDH is consistently higher than Al­(OH)_3_, indicating ongoing electrochemical activity which can be attributed to the LDH layered structure in enabling sustained current flow, likely due to lithium ion transport within the layers (Figure 4D).

LDHs rely primarily on electrostatic interactions between positively charged hydroxylated layers and the exchanged anions, with a minor contribution from the free energy associated with hydration changes.^62^ This is due to the presence of exchangeable anions and water molecules bonded within their interlayer spaces.^63^ The characterization results of Al-LDH reveal the incorporation of carbonate anions within chloride ions in its structure. The carbonates in the LDHs structure exhibit a selective affinity toward anions. The substitution of chloride ions with carbonate reduces the interlayer spacing of LDHs, thereby enhancing the binding energy between the interlayer anions and the basal layer.^11^ This structural modification decreases the interlayer gaps and improves the selectivity of the material toward elements with small ionic diameters, such as lithium.^62,64^ This selective interaction with carbonates enables a more targeted and efficient incorporation of lithium ions into the structure, thereby enhancing the overall performance of the cell. The layered structure of Al-LDH facilitates an optimal framework for the intercalation of anions between the layers. This structural arrangement preferentially permits the transport of lithium ions.^59^ In contrast, the Al­(OH)_3_ electrode does not exhibit the same level of selectivity. Its more generalized cation uptake behavior suggests that it lacks the specialized ion transport mechanisms present in Al-LDH.

The Al-LDH electrode exhibited stable, low energy consumption across cycles, averaging ∼0.5 μWh/g. In contrast, the Al­(OH)_3_ electrode showed significantly higher energy use, averaging 0.03 mWh/g per lithium ion, likely due to electrochemical inefficiencies and discharge energy losses. Elevated consumption in the first three cycles may result from the material’s high adsorptivity and complex electrochemical behavior. Cyclic voltammetry in a three-electrode setup revealed quasi-reversible behavior for Al-LDH between 0 and −0.6 V vs Ag/AgCl (Supporting Information, Figure S6A).

Although the Al­(OH)_3_ sorbent is not electrochemically active, the quasi-reversible peak of the intercalated AL-LDH material between reduction and oxidation peaks can be associated with the insertion of lithium ions into the crystal structure of Al­(OH)_3_ and crystal growth of the material, causing expansion in the hexagonal structure.^14,19^

Table 1 displays various sorption studies on lithium-layered materials. The removal efficiency typically falls within the range of 70–95%. The study indicated that the AL-LDH material exhibited a release efficiency of 53% and an uptake efficiency of around 74%. Although the release efficiency is relatively lower compared to the uptake efficiency, the process is reversible, and the electrode’s self-regeneration slows down after 15 cycles. The average regeneration ratio for the Al-LDH electrode is 35%, significantly surpassing the 29% value found for Al­(OH)_3_. These results highlight the potential of Al-LDH as a regenerable material for electrochemical lithium ion extraction; however, further optimization is required to overcome current limitations and fully realize its practical applicability.

**Table 1:** Comparison of the Al–Li Layered Materials in Different Lithium Extraction Studies

material	initial lithium ion concentration (mg/L)	lithium ion up take capacity (mg/g)	removal of lithium ions (%)	method	ref
Al(OH)_3_	1150	943.5	92–94	adsorption	^13^
Li/Al layered double hydroxides (Li/Al-LDHs)	399	7.3	72	adsorption	^22^
magnetic Li/Al-LDHs doped with Fe_3_O_4_ nanoparticles (NPs)	397	5.8		adsorption	^65^
granulated Li/Al-LDHs and NH_4_Al_3_(OH)_6_	solution 1:500	solution 1:9.7		adsorption	^66^
	solution 2:969	solution 2:9.2			^66^
granulated porous Li/Al-LDHs	solution 1:300	solution 1:8.5		adsorption	^20^
	solution 2:999	solution 2:11.8			^20^
granulated Li/Al-LDHs	1400	14.5	83	adsorption	^67^
Zn^2+^-doped Li/Al-LDHs	solution 1:750	solution 1:13.1		adsorption	^45^
	solution 2:222	solution 2:6.1			^45^
Li/Al-LDHs dopedwith Fe_3_O_4_ NPs	397.1	13.4		adsorption	^68^
LiAl-LDH beads	200–500	BLDH-P:5.3 mg/g		adsorption	^69^
		BLDH-Cl: 4.7 mg/g			^69^
LDH-Si-BX	10–25–50	1.7 mg/g	83%	adsorption	^57^
*Al-LDH*	0.4	57.6	60	*chemisorption with electrochemical regeneration*	*this work*

## 4 Conclusions

In this study, the synthesis of the Al-LDH material was successfully carried out under mild reaction conditions, ensuring an environmentally friendly and energy-efficient process. Following synthesis, the Al-LDH material′s structure has been characterized using a variety of advanced techniques to analyze its structural, morphological, and compositional properties. These techniques included X-ray diffraction for phase identification, Fourier-transform infrared spectroscopy for functional group analysis, and scanning electron microscopy for examining surface morphology. The characterization results provided a comprehensive understanding of the Al-LDH’s crystalline structure, particle size distribution, and surface properties. Our findings reveal that the Al-LDH electrode showcased the adsorption–desorption capacity for lithium ions.

The two-step process, which involves chemical sorption followed by electrochemical desorption, enabled efficient lithium ion uptake from mixed-ion solutions, with Al-LDH significantly outperforming unmodified Al­(OH)_3_ in both selectivity and capacity. The layered structure of Al-LDH facilitated high lithium ion uptake (average of 57.6 mg/g), and its selectivity factor toward lithium ions over sodium ions was approximately 2.5 times higher than that of Al­(OH)_3_. The desorption efficiency remained comparatively lower, with an average of 13 ± 3 mg/g of lithium ions released. This asymmetry is attributed to the strong electrostatic and coordinative interactions between lithium ions and the hydroxide layers, which enhance selectivity while also stabilizing intercalated ions and impeding their release. Lithium ions can be recovered by the additional step of pH-neutral washing, which is beneficial in preserving the performance of the electrode and thereby circumventing the dissolution damage to lithium-adsorbed adsorbents typically caused by acidic eluents. In particular, the electrochemical regeneration process demonstrated here proceeds under mild conditions and does not require the use of acidic eluents, making it both environmentally benign and operationally advantageous.

The layered structure of the Al-LDH material plays a crucial role by promoting the ingress of lithium ions while effectively blocking the penetration of larger sodium ions. Selectivity comparison of the materials highlights the significance of electrode material choice in applications where selective lithium ion extraction is desired. The results confirm that Al-LDH electrodes provide a more effective and sustainable solution in electrochemical systems, owing to their higher release capacity and higher purity level. The findings have promising implications for sustainable lithium ion recovery, contributing to greener energy-storage systems and paving the way for scalable, environmentally conscious extraction technologies that address global demand while reducing ecological impact.

## Supplementary Material



## Data Availability

All data can be accessed via 10.5281/zenodo.15575958.

## Notes

Additional material characterization of the electrodes: SEM and TEM of the Al­(OH)_3_; nitrogen gas sorption analysis of the Al­(OH)_3_ electrode; analytical data set of washing steps; XPS of C 1s of Al-LDH; XRD of the Al-LDH and post mortem electrode after 15 cycles; and cyclic voltammogram of the Al-LDH electrode (PDF)

## References

[1] Reich R., Slunitschek K., Danisi R. M., Eiche E., Kolb J. (2023). Lithium extraction
techniques and the application potential of different sorbents for
lithium recovery from brines. Mineral Processing
and Extractive Metallurgy Review.

[2] Jaskula, B. W. U.S. Geological survey mineral commodity summaries 2024 data release (ver. 2.0, March 2024); U.S. Geological Survey, 2024.

[3] IEA (2024). Global critical minerals outlook 2024, IEA, Paris, Licence: CC BY 4.0; IEA, International Energy Agency, 2024. https://www.iea.org/reports/global-critical-minerals-outlook-2024.

[4] Tabelin C. B., Dallas J., Casanova S., Pelech T., Bournival G., Saydam S., Canbulat I. (2021). Towards a
low-carbon society: A review
of lithium resource availability, challenges and innovations in mining,
extraction and recycling, and future perspectives. Minerals Engineering.

[5] Farahbakhsh J., Arshadi F., Mofidi Z., Mohseni-Dargah M., Kök C., Assefi M., Soozanipour A., Zargar M., Asadnia M., Boroumand Y. (2024). Direct lithium extraction: A new paradigm for lithium production
and resource utilization. Desalination.

[6] Meng F., McNeice J., Zadeh S. S., Ghahreman A. (2021). Review of
lithium production and recovery from minerals, brines, and lithium-ion
batteries. Mineral Processing and Extractive
Metallurgy Review.

[7] Boroumand Y., Razmjou A. (2024). Adsorption-type aluminium-based direct
lithium extraction:
The effect of heat, salinity and lithium content. Desalination.

[8] Wang H., Huang K., Zhang Y., Chen X., Jin W., Zheng S., Zhang Y., Li P. (2017). Recovery of lithium,
nickel, and cobalt from spent lithium-ion battery powders by selective
ammonia leaching and an adsorption separation system. ACS Sustainable Chem. Eng..

[9] Vera M. L., Torres W. R., Galli C. I., Chagnes A., Flexer V. (2023). Environmental
impact of direct lithium extraction from brines. Nature Reviews Earth & Environment.

[10] Yu J., Daliang F., Zhang H., Leong Z. Y., Zhang J., Li X., Yang H. Y. (2020). Ocean mining:
A fluidic electrochemical route for lithium
extraction from seawater. ACS Mater. Lett..

[11] Wang L., Arnold S., Ren P., Qingsong W., Jin J., Wen Z., Presser V. (2022). Redox flow
battery for continuous and energy-effective
lithium recovery from aqueous solution. ACS
Energy Lett..

[12] Ighalo J. O., Amaku J., Olisah C., Adeola A., Iwuozor K., Akpomie K., Conradie J., Adegoke K. A., Oyedotun K. (2022). Utilisation
of adsorption as a resource recovery technique for lithium in geothermal
water. J. Mol. Liq..

[13] Al-dhawi B. N. S., Kutty S. R. M., Baloo L., Alawag A. M., Almahbashi N. M. Y., Naji G. M. A., Alsaeedi Y. A. A., Al-Towayti F. A. H., Jagaba A. H. (2023). Lithium adsorption from aqueous solution using aluminum
hydroxide: Characterization, optimization by response surface methodology,
kinetic modelling, and isotherm studies. Case
Studies in Chemical and Environmental Engineering.

[14] Wang M., Zhang T., Meng Z., Wang C., Dong W., Liu J., Yang S., Hou X., Cheng X., Liu W. (2023). Self-intercepting interference
of hydrogen-bond induced flexible
hybrid film to facilitate lithium extraction. Chemical Engineering Journal.

[15] Zhang Y., Sun W., Xu R., Wang L., Tang H. (2021). Lithium extraction
from water lithium resources through green electrochemical-battery
approaches: A comprehensive review. Journal
of Cleaner Production.

[16] Jiang H., Yang Y., Sun S., Yu J. (2020). Adsorption
of lithium
ions on lithium-aluminum hydroxides: Equilibrium and kinetics. Canadian Journal of Chemical Engineering.

[17] Yu H., Naidu G., Zhang C., Wang C., Razmjou A., Han D., He T., Shon H. (2022). Metal-based adsorbents for lithium
recovery from aqueous resources. Desalination.

[18] Khalil A., Mohammed S., Hashaikeh R., Hilal N. (2022). Lithium recovery from
brine: Recent developments and challenges. Desalination.

[19] Sun Y., Yun R., Zang Y., Pu M., Xiang X. (2019). Highly efficient lithium
recovery from pre-synthesized chlorine-ion-intercalated LiAl-layered
double hydroxides via a mild solution chemistry process. Materials.

[20] Dong M., Luo Q., Li J., Wu Z., Liu Z. (2022). Lithium adsorption
properties of porous LiAl-layered double hydroxides synthesized using
surfactants. Journal of Saudi Chemical Society.

[21] Kotsupalo N. P., Ryabtsev A. D., Poroshina I. A., Kurakov A. A., Mamylova E. V., Menzheres L. T., Korchagin M. A. (2013). Effect of structure on the sorption
properties of chlorine-containing form of double aluminum lithium
hydroxide. Russian Journal of Applied Chemistry.

[22] Zhong J., Lin S., Yu J. (2021). Li^+^ adsorption performance and mechanism
using lithium/aluminum layered double hydroxides in low grade brines. Desalination.

[23] Xu X., Chen Y., Wan P., Gasem K., Wang K., He T., Adidharma H., Fan M. (2016). Extraction of lithium with functionalized
lithium ion-sieves. Prog. Mater. Sci..

[24] Martínez-Rodríguez M. J., García-Díaz B. L., Teprovich J. A., Knight D. A., Zidan R. (2012). Advances in the electrochemical regeneration
of aluminum hydride. Appl. Phys. A: Mater. Sci.
Process..

[25] Srimuk P., Husmann S., Presser V. (2019). Low voltage operation
of a silver/silver
chloride battery with high desalination capacity in seawater. RSC Adv..

[26] Kök C., Wang L., Ruthes J. G. A., Quade A., Suss M. E., Presser V. (2024). Continuous Lithium-Ion
Extraction From Seawater and
Mine Water With a Fuel Cell System and Ceramic Membranes. Energy&Environmental Materials.

[27] Zavahir S., Elmakki T., Gulied M., Ahmad Z., Al-Sulaiti L., Shon H. K., Chen Y., Park H., Batchelor B., Han D. S. (2021). A review on lithium
recovery using electrochemical
capturing systems. Desalination.

[28] Munk L. A., Hynek S. A., Bradley D. C., Boutt D., Labay K., Jochens H. (2016). Lithium Brines: A Global
Perspective. Journal of Economic Geology.

[29] Paranthaman M. P., Li L., Luo J., Hoke T., Ucar H., Moyer B. A., Harrison S. (2017). Recovery of
lithium from geothermal brine with lithium-aluminum
layered double hydroxide chloride sorbents. Environ. Sci. Technol..

[30] Cheary R. W., Coelho A. (1992). A fundamental parameters approach to X-ray line-profile
fitting. J. Appl. Crystallogr..

[31] Zeiger M., Jäckel N., Weingarth D., Presser V. (2015). Vacuum or flowing argon:
What is the best synthesis atmosphere for nanodiamond-derived carbon
onions for supercapacitor electrodes?. Carbon.

[32] Gor G., Thommes M., Cychosz K., Neimark A. (2012). Quenched solid density
functional theory method for characterization of mesoporous carbons
by nitrogen adsorption. Carbon.

[33] Torkamanzadeh M., Kök C., Burger P. R., Ren P., Zhang Y., Lee J., Kim C., Presser V. (2023). Best practice for electrochemical
water desalination data generation and analysis. Cell Reports Physical Science.

[34] Ewert J. K., Weingarth D., Denner C., Friedrich M., Zeiger M., Schreiber A., Jäckel N., Presser V., Kempe R. (2015). Enhanced capacitance
of nitrogen-doped
hierarchically porous carbide-derived carbon in matched ionic liquids. Journal of Materials Chemistry A.

[35] Kim C., Srimuk P., Lee J., Fleischmann S., Aslan M., Presser V. (2017). Influence of pore structure
and cell
voltage of activated carbon cloth as a versatile electrode material
for capacitive deionization. Carbon.

[36] Venkataraman K., Pachayappan L. (2020). Synthesis
of nordstrandite and nordstrandite-derived
layered double hydroxides of Li and Al: A comparative study with the
bayerite counterpart. Zeitschrift für
anorganische und allgemeine Chemie.

[37] Besserguenev A. V., Fogg A. M., Francis R. J., Price S. J., O’Hare D., Isupov V. P., Tolochko B. P. (1997). Synthesis
and structure of the Gibbsite
intercalation compounds [LiAl_2_(OH)_6_]­X {X = Cl,
Br, NO_3_} and [LiAl_2_(OH)_6_]­Cl·H_2_O using synchrotron X-ray and neutron powder diffraction. Chem. Mater..

[38] Britto S., Joseph S., Vishnu Kamath P. (2010). Distinguishing crystallite size effects
from those of structural disorder on the powder X-ray diffraction
patterns of layered materials. Journal of Chemical
Sciences.

[39] Britto S., Kamath P. V. (2012). Structural synthon approach to the
study of stacking
faults in the layered double hydroxides of lithium and aluminum. Zeitschrift für anorganische und allgemeine Chemie.

[40] Mata D., Serdechnova M., Mohedano M., Mendis C. L., Lamaka S. V., Tedim J., Hack T., Nixon S., Zheludkevich M. L. (2017). Hierarchically
organized Li–Al-LDH nano-flakes: a low-temperature approach
to seal porous anodic oxide on aluminum alloys. RSC Adv..

[41] Bhuvaneswari K., Palanisamy G., Pazhanivel T., Maiyalagan T., Bharathi G. (2019). Photodegradation activity of nitrogen-rich graphitic
carbon nitride intercalated ZnO Mg-Al layered double hydroxide ternary
nanocomposites on methylene blue dye. ChemistrySelect.

[42] Yuan X., Yin C., Zhang Y., Chen Z., Xu Y., Wang J. (2019). Synthesis
of C@Ni-Al LDH HSS for efficient U-entrapment from seawater. Sci. Rep..

[43] Punnakkal N., Jayakanthan S., Kumar M., P A.
K., Pradeep A., P V S., Satheesh Babu T. G. (2024). High surface area cobalt aluminium
layered double hydroxide printed electrodes for flexible supercapacitor
and on-chip electrochemical bacterial lysing. Electrochim. Acta.

[44] Thommes, M. ; Guillet-Nicolas, R. ; Cychosz, K. Physical adsorption characterization of mesoporous zeolites, 2015; pp 349–384.

[45] Zhang L., Zhang T., Zhao Y., Dong G., Lv S., Ma S., Song S., Quintana M. (2024). Doping engineering of lithium-aluminum
layered double hydroxides for high-efficiency lithium extraction from
salt lake brines. Nano Research.

[46] Flores E., Novák P., Berg E. J. (2018). In situ and operando Raman spectroscopy
of layered transition metal oxides for Li-ion battery cathodes. Front. Energy Res..

[47] Ruther R., Callender A., Zhou H., Martha S., Nanda J. (2015). Raman microscopy
of lithium-manganese-rich transition metal oxide cathodes. J. Electrochem. Soc..

[48] Mindivan H., Sabri Kayali E., Cimenoglu H. (2008). Tribological behavior of squeeze
cast aluminum matrix composites. Wear.

[49] Revo S., Hamamda S., Ivanenko K., Boshko O., Djarri A., Boubertakh A. (2015). Thermal analysis of Al + 0.1% CNT ribbon. Nanoscale Res. Lett..

[50] Sun Q., Qin C. (2011). Raman OH stretching band of water as an internal standard
to determine
carbonate concentrations. Chem. Geol..

[51] Presser V., Kloužková A., Mrázová M., Kohoutková M., Berthold C. (2008). Micro-Raman spectroscopy on analcime
and pollucite in comparison to X-ray diffraction. J. Raman Spectrosc..

[52] Rudolph W. W., Hefter G. T. (2009). Quantitative analysis in alkaline aluminate solutions
by Raman spectroscopy. Analytical Methods.

[53] Moulder, J. F. ; Chastain, J. Handbook of X-ray photoelectron spectroscopy: A reference book of standard spectra for identification and interpretation of XPS data; Physical Electronics Division, Perkin-Elmer Corporation, 1992.

[54] Visser P., Lutz A., Mol J. M. C., Terryn H. (2016). Study of the formation
of a protective layer in a defect from lithium-leaching organic coatings. Prog. Org. Coat..

[55] Barik S., Badamali S., Behera L., Jena P. (2018). Mg–Al LDH reinforced
PMMA nanocomposites: a potential material for packaging industry. Compos. Interfaces.

[56] Theiss F. L., Ayoko G. A., Frost R. L. (2013). Thermogravimetric
analysis of selected
layered double hydroxides. J. Therm. Anal. Calorim..

[57] Qian C., Zheng M., Zhang Y., Xing E., Gui B. (2023). Adsorption
performance and mechanism of Li+ from brines using lithium/aluminum
layered double hydroxides-SiO2 bauxite composite adsorbents. Frontiers in Chemistry.

[58] Wu L., Li L., Evans S. F., Eskander T. A., Moyer B. A., Hu Z., Antonick P. J., Harrison S., Paranthaman M. P., Riman R. (2019). Lithium aluminum-layered double hydroxide chlorides
(LDH): Formation enthalpies and energetics for lithium ion capture. J. Am. Ceram. Soc..

[59] Guo Y., Yu J., Su H., Lin S. (2024). Desorption enhancement of aluminum-based
adsorbent in lithium extraction from sulfate-type salt lakes. Desalination.

[60] Gao Y., Wu J., Zhang Z., Jin R., Zhang X., Yan X., Umar A., Guo Z., Wang Q. (2013). Synthesis of polypropylene/Mg_3_Al–X (X = CO_3_
^2–^, NO_3_
^–^, Cl^–^, SO_4_
^2–^) LDH nanocomposites using a solvent mixing method:
thermal and melt rheological properties. Journal
of Materials Chemistry A.

[61] Lv S., Zhao Y., Zhang L., Zhang T., Dong G., Li D., Cheng S., Ma S., Song S., Quintana M. (2023). Anion regulation
strategy of lithium-aluminum layered double hydroxides for strengthening
resistance to deactivation in lithium recovery from brines. Chemical Engineering Journal.

[62] Forano C., Hibino T., Leroux F., Taviot-Gueho C. (2006). Layered double
hydroxides. Developments in Clay Science.

[63] Dékány I., Berger F., Imrik K., Lagaly G. (1997). Hydrophobic layered
double hydroxides (LDHs): Selective adsorbents for liquid mixtures. Colloid & Polymer Science.

[64] Razmjou A., Asadnia M., Hosseini E., Habibnejad Korayem A., Chen V. (2019). Design principles of ion selective
nanostructured membranes for the
extraction of lithium ions. Nat. Commun..

[65] Chen J., Lin S., Yu J. (2020). Quantitative
effects of Fe_3_O_4_ nanoparticle content on Li^+^ adsorption and magnetic recovery
performances of magnetic lithium-aluminum layered double hydroxides
in ultrahigh Mg/Li ratio brines. Journal of
Hazardous Materials.

[66] Luo Q., Mingzhe D., Nie G., Liu Z., Wu Z., Li J. (2021). Extraction of lithium from salt lake brines by granulated adsorbents. Colloids Surf., A.

[67] Li X., Chen L., Chao Y., Zhu L., Luo G., Sun J., Jiang L., Zhu W., Liu Z., Xu C. (2022). Highly selective
separation of lithium with hierarchical porous lithium-ion sieve microsphere
derived from MXene. Desalination.

[68] Chen J., Lin S., Yu J. (2021). High-selective
cyclic adsorption and magnetic recovery
performance of magnetic lithium-aluminum layered double hydroxides
(MLDHs) in extracting Li^+^ from ultrahigh Mg/Li ratio brines. Sep. Purif. Technol..

[69] Zhang L., Zhang T., Lv S., Cheng S., Dong G., Quintana M., Song S., Zhao Y. (2024). Steering interlayer
interaction of lithium-aluminum layered double hydroxide beads for
stable lithium extraction from sulfate-type brines. Desalination.

