# Correction to “Acid-Free Electrochemical Regeneration of Sandrose-like Aluminum Layered Double Hydroxide Electrodes for Selective Lithium-Ion Recovery in Mixed Ion Solution”

**DOI:** 10.1021/acssuschemeng.5c13469

**Published:** 2025-12-26

**Authors:** Cansu Kök, Pablo Vega Hernández, Jean G. A. Ruthes, Oliver Janka, Antje Quade, Volker Presser

**Affiliations:** † INM - Leibniz Institute for New Materials, Campus D2 2, 66123 Saarbrücken, Germany; ‡ Department of Materials Science & Engineering, Saarland University, Campus D2 2, 66123 Saarbrücken, Germany; § saarene - Saarland Center for Energy Materials and Sustainability, Campus C4 2, 66123 Saarbrücken, Germany; ∥ Inorganic Solid State Chemistry, Saarland University, Campus C4 1, 66123 Saarbrücken, Germany; ⊥ 28372 Leibniz Institute for Plasma Science and Technology, Felix-Hausdorff-Straße 2, 17489 Greifswald, Germany

In the original Article, Figure 1C shows an image from a different, morphologically similar sample rather than the sample investigated in the other panels and throughout the manuscript. A corrected version of Figure 1 (with the correct image for panel C) is provided below. This correction does not affect the results, discussion, or conclusions of the Article.

**Figure 1:**
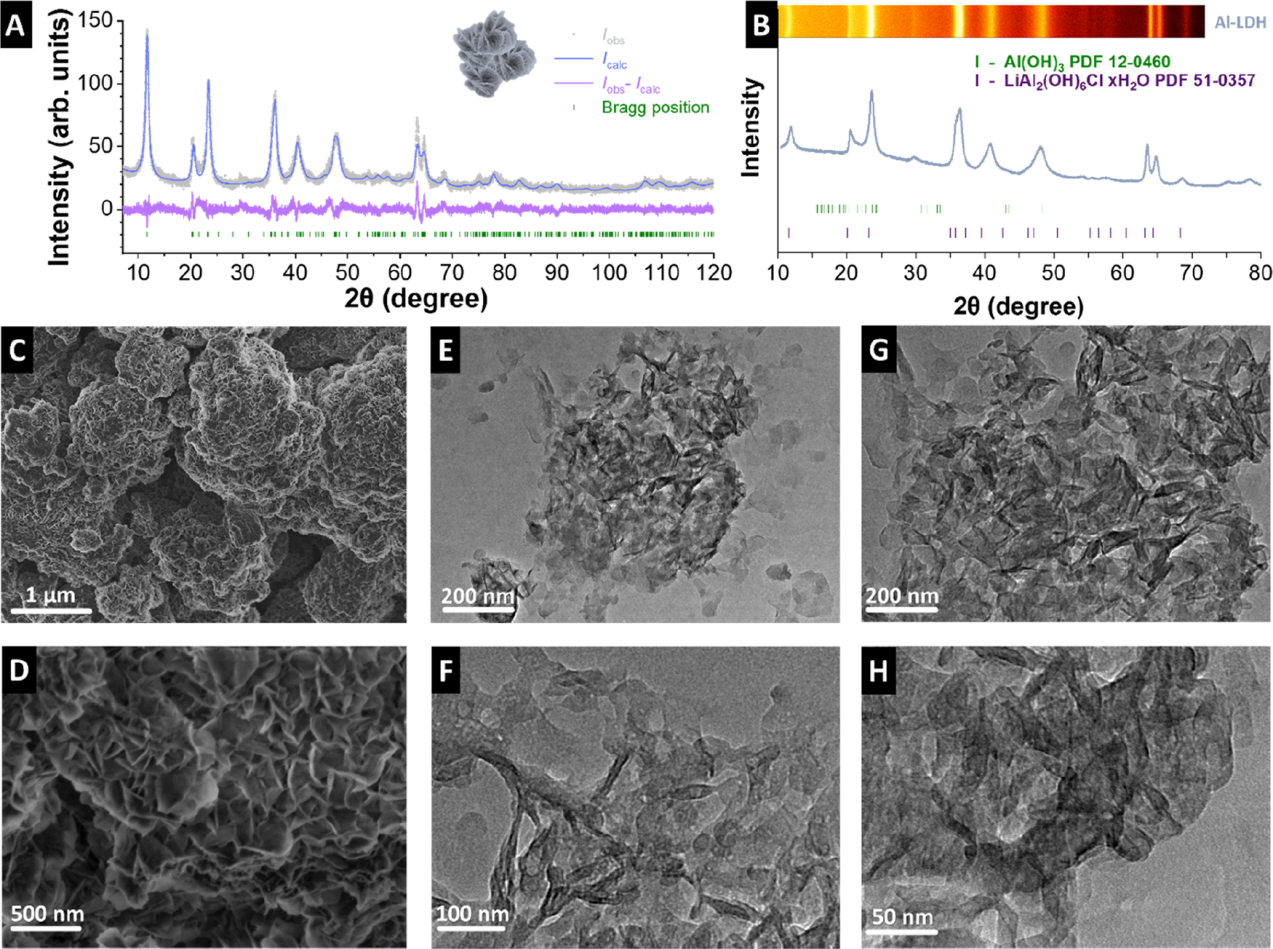
(A) X-ray diffractogram and associated Rietveld fitting of Al-LDH. (B) Normalized X-ray diffractograms of Al-LDH. (C and D) Scanning electron micrographs of Al-LDH at different magnifications. (E–H) Transmission electron micrographs of Al-LDH.

