# Supplementary material for: Acid-Free Electrochemical Regeneration of Sandrose-like Aluminum Layered Double Hydroxide Electrodes for Selective Lithium-Ion Recovery in Mixed Ion Solution
Source: ACS Sustain Chem Eng. 2025 Oct 31;13(44):19218–28. doi: 10.1021/acssuschemeng.5c08261 (PMC12610405; doi:10.1021/acssuschemeng.5c08261)
Supplement: Supplementary file 1 [file sc5c08261_si_001.pdf]

Supporting Information

Acid-Free Electrochemical Regeneration of Sandrose-like Aluminum Layered Double Hydroxide Electrodes for Selective Lithium-Ion Recovery in Mixed Ion Solution

Cansu Kök,[1,2] Pablo Vega Hernandez,[1,2] Jean G. A. Ruthes,[1,2]
Oliver Janka,[4] Antje Quade,[5] Volker Presser,[1,2,3]*

[1] *INM - Leibniz Institute for New Materials, Campus D2 2, 66123, Saarbrücken, Germany*

[2] *Department of Materials Science & Engineering, Saarland University, Campus D2 2, 66123, Saarbrücken, Germany*

[3] *saarene - Saarland Center for Energy Materials and Sustainability, Campus C4 2, 66123 Saarbrücken, Germany*

[4] *Inorganic Solid State Chemistry, Saarland University, Campus C4 1, 66123, Saarbrücken, Germany*

[5] *Leibniz Institute for Plasma Science and Technology, Felix-Hausdorff-Straße 2, 17489, Greifswald, Germany*

* Corresponding author: volker.presser@leibniz-inm.de (VP)

Content (5 pages):

Additional material characterization of the electrodes: SEM and TEM of the $Al(OH)_3$. Nitrogen gas sorption analysis of the $Al(OH)_3$ electrode. Analytical dataset of washing stages. XPS of C1s of Al-LDH. XRD of the Al-LDH and post mortem electrode after 15 cycles. Cyclic voltammogram of the Al-LDH electrode.

1. Supplementary Figures

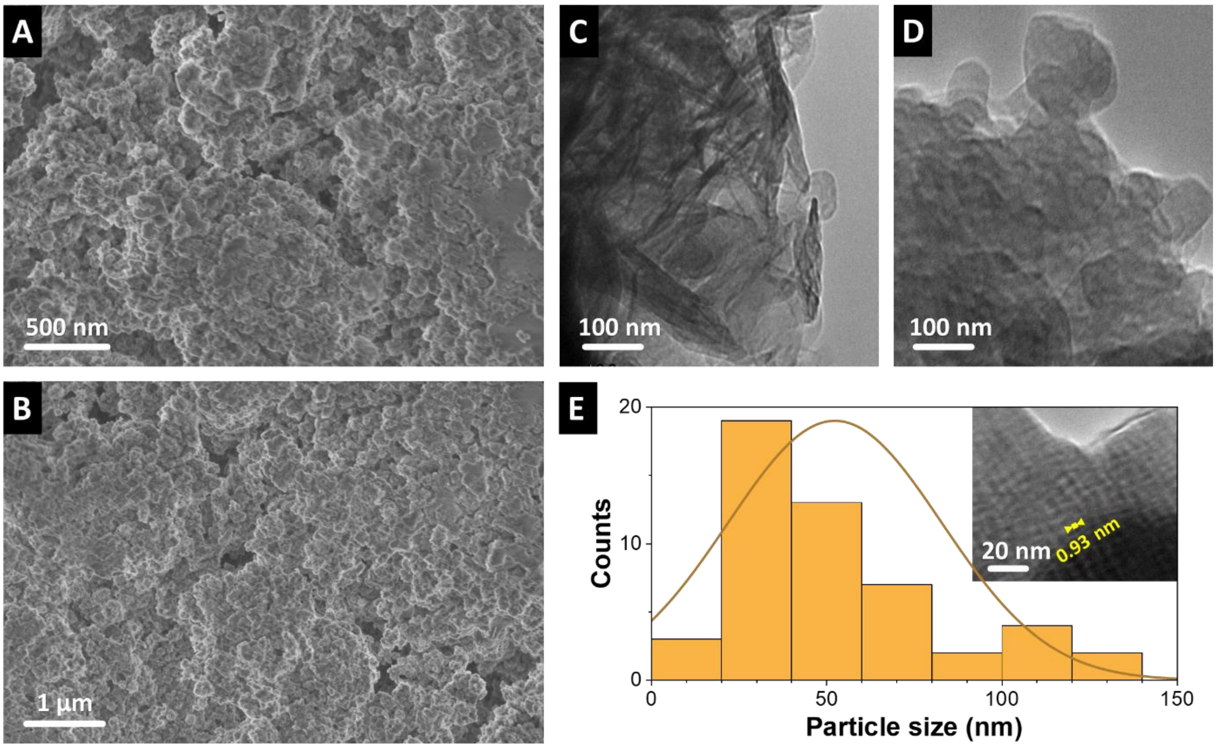

Fig. S1. A-B) Scanning electron micrographs**, C-D)** Transmission electron micrographs, **E)** average particle size of the $Al(OH)_3$ of 60 counts.

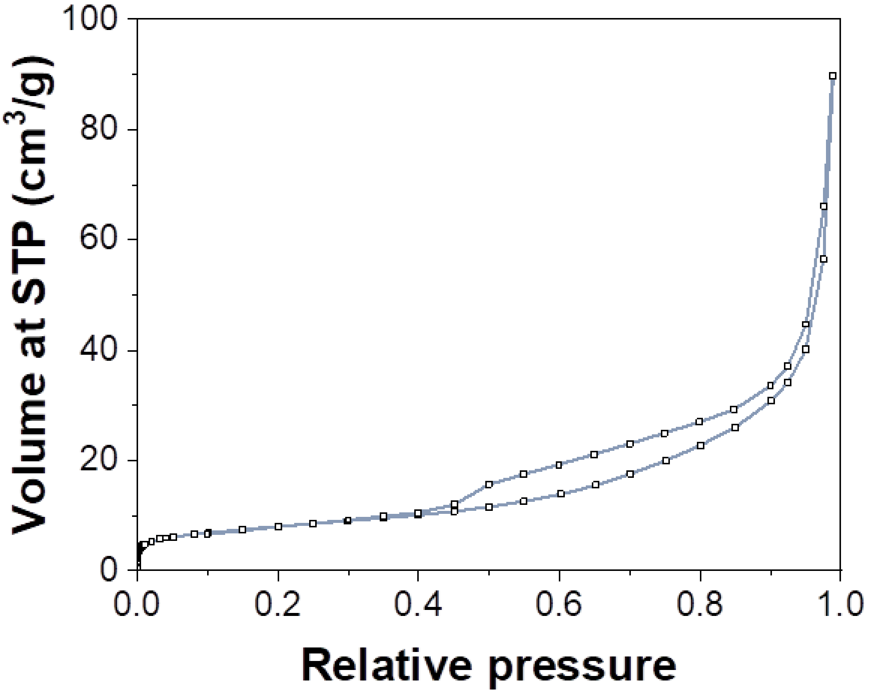

Fig. S2. Nitrogen gas sorption isotherm recorded at -196 °C of the $Al(OH)_3$ electrode.(STP: standard temperature and pressure).

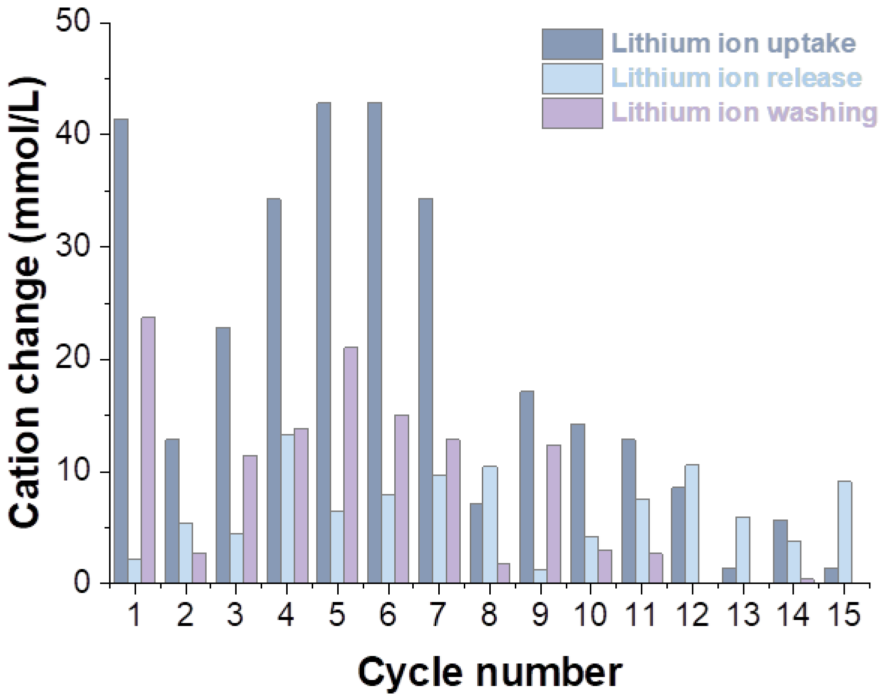

Fig. S3. Comparison of lithium ion amount after uptake, release, and washing steps.

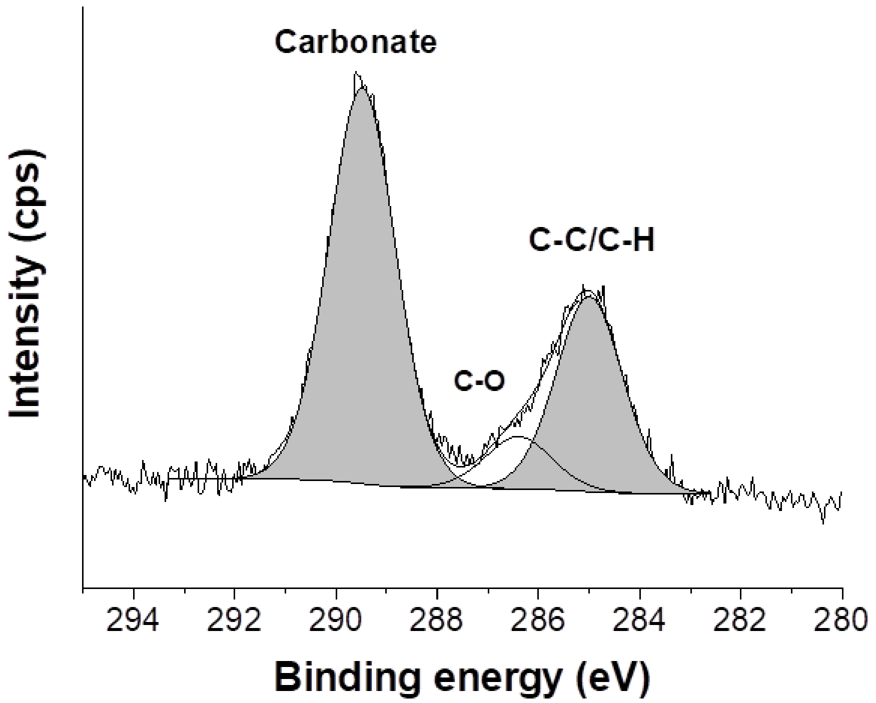

Fig. S4. X-ray photoelectron spectrum of C 1s of Al-LDH.

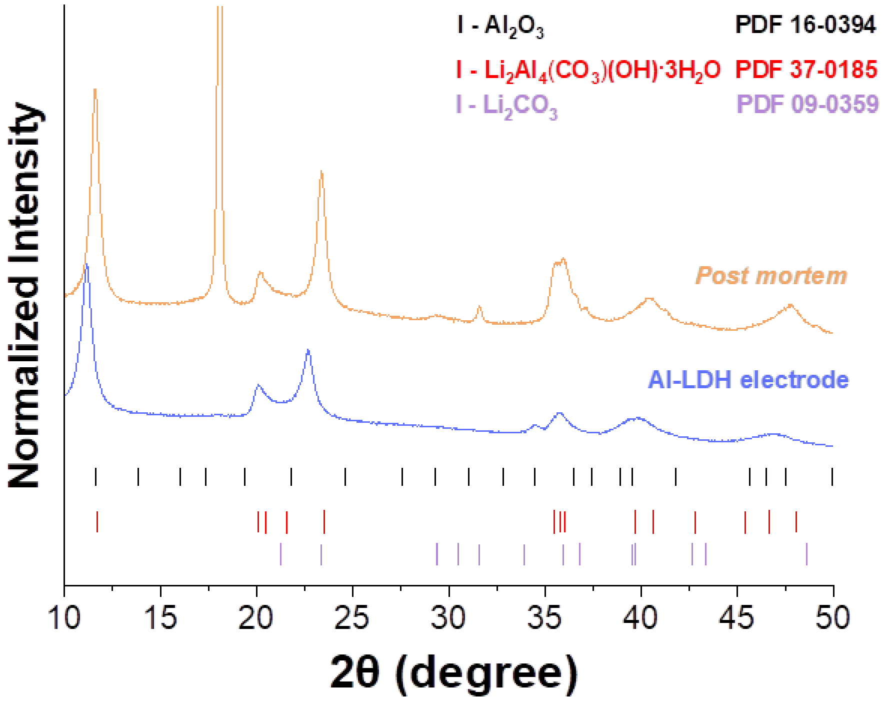

Fig. S5. X-ray diffractograms of the Al-LDH and post mortem electrode after 15 cycles.

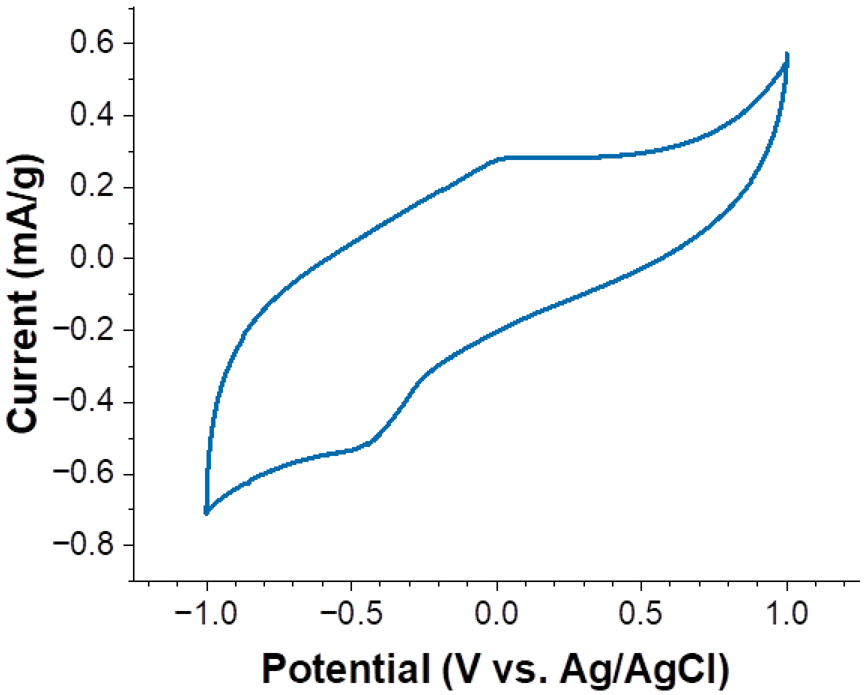

Fig. S6. Cyclic voltammogram of the Al-LDH electrode at 10 mV/s in 1 M LiCl.
